# Stepwise Algorithm for Biliary Cannulation in Endoscopic Retrograde Cholangiopancreatography in the Era of Endoscopic Ultrasound‐Guided Biliary Drainage: A Single‐Center Retrospective Cohort Study

**DOI:** 10.1002/deo2.70272

**Published:** 2026-01-06

**Authors:** Akihiko Senju, Takuji Iwashita, Takuya Koizumi, Yosuke Ohashi, Shota Iwata, Akinori Maruta, Shinya Uemura, Masahito Shimizu

**Affiliations:** ^1^ First Department of Internal Medicine Gifu University Hospital Gifu Japan; ^2^ Department of Gastroenterology Seino Kosei Hospital Gifu Japan; ^3^ Department of Gastroenterology Shiga University of Medical Science Shiga Japan

**Keywords:** difficult biliary cannulation, double‐guidewire technique, endoscopic ultrasound‐guided rendezvous technique (EUS‐RV), precut technique, post‐ERCP pancreatitis

## Abstract

**Introduction:**

Endoscopic retrograde cholangiopancreatography (ERCP) is an essential procedure for the management of biliary diseases. Successful biliary cannulation is the first step. Advanced cannulation techniques, such as the double‐guidewire technique (DGW), precut techniques, and endoscopic ultrasound (EUS)‐guided rendezvous (RV), have been developed to improve cannulation success. However, comprehensive evaluations of a structured cannulation algorithm remain limited.

**Aims:**

To evaluate the status of biliary cannulation and associated adverse events (AEs) using a stepwise cannulation algorithm.

**Methods:**

A retrospective evaluation of 1,000 consecutive patients with a naïve papilla who underwent ERCP for biliary disease was performed between 2012 and 2022. Biliary cannulation was attempted using a stepwise algorithm, beginning with wire‐loaded cannulation (WLC), followed by DGW, precut techniques, and EUS‐RV. The primary endpoint was overall technical success; secondary endpoints included AEs, incidence of post‐ERCP pancreatitis (PEP), and risk factor analysis.

**Results:**

Initial WLC achieved selective biliary cannulation in 69.2% of cases. Salvage techniques achieved high success rates: DGW (74.9%), precut techniques (77.6%), and EUS‐RV (97.0%). Overall, the final cannulation success rate was 97.8%. The overall AE rate was 7.5%, with PEP being the most common (6.1%). AEs were significantly more frequent in advanced cannulation techniques than WLC (13.3% vs. 5.2%, *p* < 0.001). Multivariate analysis identified advanced cannulation techniques, pancreatography, and metallic stent placement as independent factors increasing the risk of PEP.

**Conclusion:**

A structured stepwise approach achieves very high biliary cannulation success in patients with a naïve papilla, though advanced cannulation techniques increase AE risk. Appropriate timing and positioning of EUS‐RV may further optimize safety and efficacy in biliary cannulation.

**Abbreviations**
AE: adverse event
CI: confidence interval
DGW: double‐guidewire technique
ERCP: endoscopic retrograde cholangiopancreatography
EUS‐BD: endoscopic ultrasound‐guided biliary drainage
EUS‐RV: endoscopic ultrasound‐guided rendezvous technique
NKP: needle‐knife precutting
OR: odds ratios
PEP: post‐ERCP pancreatitis
PTBD: percutaneous transhepatic biliary drainage
TPS: transpancreatic precut sphincterotomy
WLC: wire‐loaded cannulation.

## 1 Background

Endoscopic retrograde cholangiopancreatography (ERCP) plays a pivotal role in both the diagnosis and treatment of biliary diseases. Diagnostic ERCP provides direct cholangiographic visualization, allowing accurate evaluation of lesions such as stones, strictures, and tumors, and enables histological confirmation through brush cytology or forceps biopsy. Therapeutically, ERCP facilitates minimally invasive interventions, including biliary drainage for obstructive jaundice or cholangitis, stone extraction, stricture dilation, and stent placement. Because ERCP allows immediate minimally invasive therapy following diagnostic assessment, it has become an essential modality for the comprehensive management of biliary disorders.

Successful biliary cannulation is the first and most essential step in ERCP; however, it is not always easily achieved. Anatomical variations, tumor infiltration, inflammatory changes, and technical limitations can hinder cannulation, thereby limiting procedural success. To improve the cannulation success rate, particularly in technically challenging cases, advanced techniques such as the double‐guidewire method and precutting have been reported as useful salvage strategies [1]. More recently, endoscopic ultrasound‐guided biliary drainage (EUS‐BD) has emerged as an alternative to ERCP‐guided drainage [2, 3]. Among its techniques, the EUS‐guided rendezvous (EUS‐RV) approach—where cannulation is facilitated by a guidewire placed under EUS guidance—has also been reported as an effective salvage method for difficult biliary cannulation. Although the efficacy and safety of each technique have been evaluated in numerous studies, most reports have focused on single salvage methods or small cohorts [4]. Comprehensive evidence regarding the overall outcomes and risks of a structured stepwise algorithm for biliary cannulation in patients with a naïve papilla remains limited.

Therefore, this study aimed to evaluate a stepwise algorithm for biliary cannulation and associated adverse events (AEs) in a large cohort of patients with a naïve papilla undergoing therapeutic ERCP for biliary disease.

## 2 Patients and Methods

### 2.1 Patient Selection

This retrospective cohort study was conducted at a single academic center, Gifu University Hospital, Japan. A prospectively maintained database of all ERCP and EUS procedures performed between April 2012 and November 2022 was reviewed to identify patients who met the following inclusion criteria: (1) underwent therapeutic ERCP for biliary disease. Exclusion criteria were: (1) a non‐naïve papilla (history of prior ERCP), (2) an ERCP indication related to pancreatic disease, (3) a history of upper gastrointestinal surgery other than Billroth I reconstruction, or (4) an endoscopically inaccessible papilla. Written informed consent was obtained from all patients prior to the procedure. The study was conducted in accordance with the ethical principles of the Declaration of Helsinki, and the protocol was approved by the Institutional Review Board of Gifu University Hospital (approval No. 2025–179).

### 2.2 Periprocedural Management

All ERCP procedures were performed on an inpatient basis. Blood tests—including complete blood counts, serum amylase, liver function tests, and renal function tests—were routinely obtained before the procedure, 2 h after, and on the morning of the following day. Patients fasted on the day of ERCP with intravenous fluids, and oral intake was resumed the next day if no AEs were detected. Prophylactic cefoperazone was routinely administered before and after the procedure. Nonsteroidal anti‐inflammatory drugs were not used for the prevention of PEP. ERCP was performed under moderate sedation with midazolam and pentazocine, with continuous monitoring of vital signs.

### 2.3 Biliary Cannulation Algorithm

**FIGURE 1:**
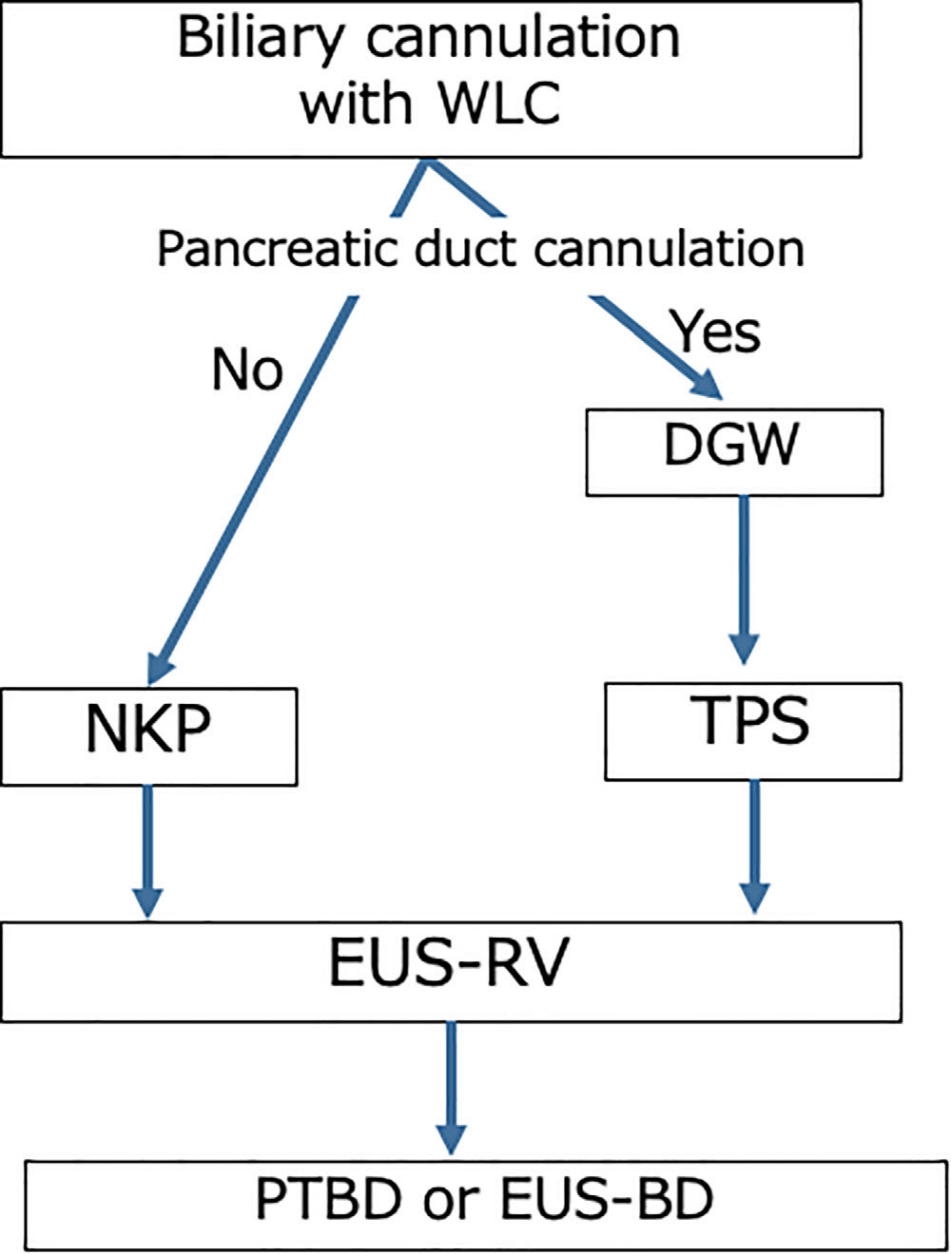
Stepwise cannulation algorithm. DGW, double‐guidewire technique; EUS‐BD, endoscopic ultrasound‐guided biliary drainage; EUS‐RV, endoscopic ultrasound‐guided rendezvous technique; NKP, needle‐knife precutting; PTBD, percutaneous trans‐hepatic biliary drainage; TPS, transpancreatic precut sphincterotomy; WLC, wire‐loaded cannulation.

**FIGURE 2:**
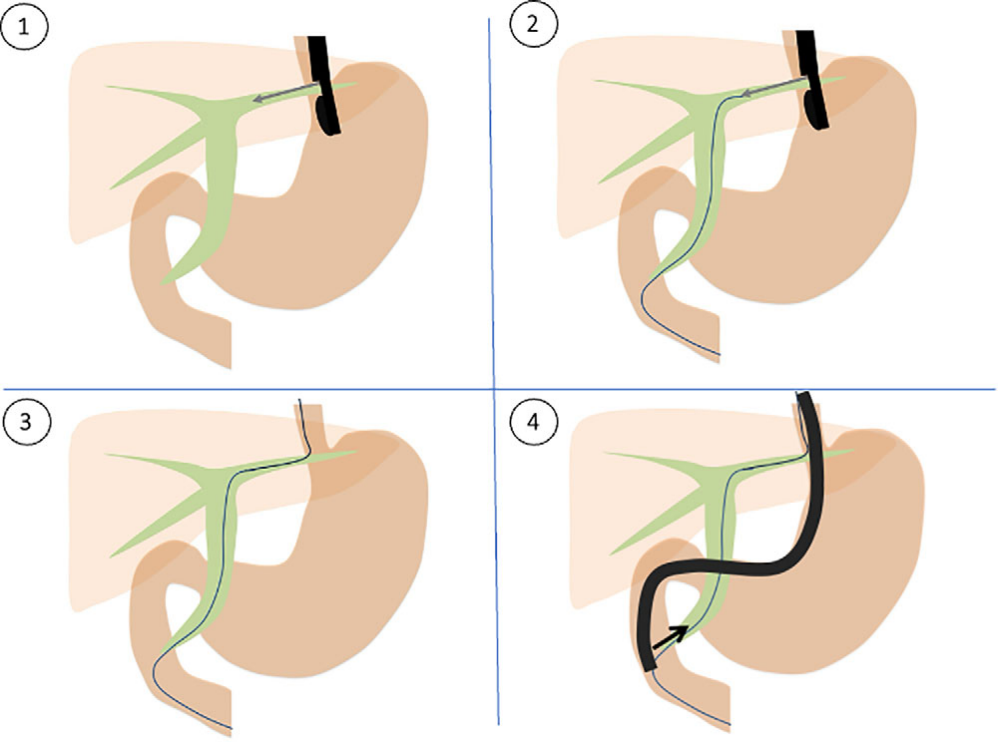
Flow of endoscopic ultrasound (EUS)‐guided rendezvous technique. 1) Bile duct is punctured under EUS guidance. 2) A guidewire is placed into the duodenum through a needle, biliary duct, and ampulla. 3) EUS is exchanged for the duodenoscope while maintaining the guidewire position. 4) Biliary cannulation is again attempted with the help of the EUS‐placed guidewire.

Bile duct cannulation was initiated with wire‐loaded cannulation (WLC) using an ERCP catheter and guidewire. If pancreatic duct cannulation was achieved but biliary cannulation failed, the double‐guidewire technique (DGW) was employed, in which a second guidewire was inserted while the first remained in the pancreatic duct. If DGW failed, transpancreatic precut sphincterotomy (TPS) was performed over the pancreatic duct guidewire using a sphincterotome. If neither biliary nor pancreatic duct cannulation was achieved, needle‐knife precutting (NKP) was attempted by making an incision in the ampulla to expose the biliary orifice. In the cases where trainees initiated the procedure, the operator was typically changed to a supervising physician when the procedure became difficult, typically after approximately 15 min of attempted cannulation, depending on the clinical situation. If all approaches failed, EUS‐RV was performed when clinically indicated and only by attending physicians who were experts in both ERCP and EUS‐related procedures. (Figure 1) In EUS‐RV, the ERCP scope was exchanged for a convex echoendoscope, the biliary system was punctured with a fine‐needle aspiration needle, and a guidewire was advanced into the intestine through the bile duct and the ampulla. The echoendoscope was then replaced with the ERCP scope, and cannulation was completed over the pre‐positioned guidewire as we previously reported [5, 6, 7] (Figure 2).

### 2.4 Definitions

Technical success was defined as successful selective biliary cannulation. AEs were classified according to the American Society for Gastrointestinal Endoscopy (ASGE) lexicon for endoscopic AEs [8]. Post‐ERCP pancreatitis (PEP) was defined as persistent abdominal pain after ERCP with a serum amylase level ≥3 times the upper limit of normal measured more than 24 h after ERCP [9].

### 2.5 Study Outcomes and Statistical Analysis

The primary outcome was the overall technical success rate of selective bile duct cannulation. Secondary outcomes included the technical success rate of each salvage technique, the overall AE rate, the incidence of PEP, and risk factors for PEP.

Continuous variables were expressed as median (range) and compared using the Mann–Whitney U test. Categorical variables were compared using Fisher's exact test. Independent risk factors for PEP were identified using multiple logistic regression, with odds ratios (ORs) and 95% confidence intervals (CIs) reported. A *p*‐value <0.05 was considered statistically significant. Analyses were performed using JMP software version 18.0 (JMP Statistical Discovery, Cary, NC, USA).

## 3 RESULTS

### 3.1 Patient Selection and Baseline Characteristics

**FIGURE 3:**
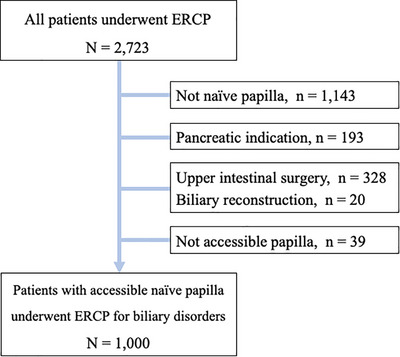
Flowchart of 2,723 patients who underwent endoscopic retrograde cholangiopancreatography.

The database analysis identified a total of 2,723 patients who underwent ERCP during the study period. After applying the inclusion and exclusion criteria, 1,000 patients with a naïve papilla who underwent ERCP for biliary disease were included in the analysis (Figure 3). Baseline characteristics of the study cohort are summarized in Table 1. The median age was 72 years (range, 11–102 years), and 56.7% were male. The most common indication for ERCP was malignant biliary stricture (47.2%), followed by bile duct stones (44.0%). Among malignant strictures, pancreatic cancer (18.9%) was the most frequent etiology, followed by bile duct cancer (11.2%), metastatic cancer (8.7%), gallbladder cancer (2.0%), and papillary cancer (1.4%). Other indications accounted for 4.5% of cases. The majority of patients had normal anatomy (98.2%), whereas 1.8% had undergone Billroth I gastrectomy.

**TABLE 1:** Baseline characteristics of the study population.

	*N* = 1,000
Age, years, median (range)	72 (11–102)
Gender, male, *n* (%)	567 (56.7)
Indication for ERCP, *n* (%)	
Bile duct stone	440 (44.0)
Cholecystitis	34 (3.4)
Benign biliary duct stricture	54 (5.4)
Malignant biliary stricture	427 (42.7)
Pancreatic cancer	189 (18.9)
Bile duct cancer	117 (11.2)
Metastatic cancer	87 (8.7)
Gallbladder cancer	20 (2.0)
Ampullary cancer	14 (1.4)
Others	45 (4.5)
Anatomy, *n* (%)	
Normal	982 (98.2)
Billroth I	18 (1.8)

### 3.2 Procedural Outcomes of ERCP

Initial WLC was successful in 69.2% (692/1,000) of patients. In cases where biliary cannulation failed but pancreatic duct cannulation was achieved, DGW achieved a success rate of 74.9% (194/259). Precut techniques had a success rate of 77.6% (59/76), including TPS of 78.8% (41/52) and NKP of 75.0% (18/24). Among the 34 patients in whom EUS‐RV was attempted, biliary cannulation was successful in 33 patients with a success rate of 97.0%, and EUS‐RV was performed during the first ERCP session in 21 of 34 patients (61.8%). Overall, the final biliary cannulation success rate was 97.8% (978/1,000). Rescue procedures included percutaneous transhepatic biliary drainage (PTBD) in six patients and EUS‐BD other than EUS‐RV in five patients, and 11 patients were managed conservatively without further intervention (Table 2).

**TABLE 2:** Outcomes of biliary cannulation using the stepwise algorithm.

	Cannulation success rates at each step, % (*n*)		Cumulative cannulation success rates, % (*n*)	
WLC	69.2%	(692/1,000)	69.2%	(692/1,000)
DGW	74.9%	(194/259)	88.6%	(886/1,000)
Precut	77.6%	(59/76)	94.5%	(945/1,000)
TPS 78.8% (41/52)				
NKP 75.0% (18/24)				
EUS‐RV	97.0%	(33/34)	97.8%	(978/1,000)
Overall cannulation success rate 97.8% (978/1000)				
Rescue procedures	PTBD in six, EUS‐BD in five, and conservative management in 11			

Abbreviations: DGW, double guidewire technique; EUS‐BD, endoscopic ultrasound‐guided biliary drainage; EUS‐RV, endoscopic ultrasound‐guided rendezvous technique; NKP, needle‐knife precutting; PTBD, percutaneous transhepatic biliary drainage; TPS, transpancreatic precut sphincterotomy; WLC, wire‐loaded cannulation.

### 3.3 Adverse Events

The overall AE rate was 7.5% (75/1,000). PEP occurred in 61 patients, bleeding in 11, perforation in two, and bile peritonitis in one. The AE rate varied by technique: WLC, 5.2% (36/692); DGW, 12.8% (25/194); precut techniques, 16.9% (10/59) [TPS, 17.1% (7/41) and NKP, 16.7% (3/18)] and EUS‐RV, 9.0% (3/33). In failed cannulation cases (*n* = 22), one patient experienced PEP (4.5%). When comparing WLC (5.2%, 36/692) with advanced cannulation techniques (13.3%, 38/286), the AE rate was significantly higher with advanced techniques (*p* < 0.001). (Table 3)

**TABLE 3:** The adverse event rates associated with each cannulation technique.

	Adverse event rate, % (*n*)		PEP, *n*	Bleeding, *n*	Perforation, *n*	Bile peritonitis, *n*
WLC	5.2 (36/692)	^*^	27	8	1	0
DGW	12.8 (25/194)	13.3 (38/286)	24	0	1	0
Precut	16.9 (10/59)		8	2	0	0
TPS 17.1% (7/41)						
NKP 16.7% (3/18)						
EUS‐RV	9.0 (3/33)		1	1	0	1
Failed cases	4.5 (1/22)		1	0	0	0
Overall	7.5 (75/1,000)		61	11	2	1

Abbreviations: DGW, double guidewire technique; EUS‐BD, endoscopic ultrasound‐guided biliary drainage; EUS‐RV, endoscopic ultrasound‐guided rendezvous technique; NKP, needle‐knife precutting; PTBD, percutaneous transhepatic biliary drainage; TPS, transpancreatic precut sphincterotomy; WLC, wire‐loaded cannulation.

* Statistically significant.

### 3.4 Risk Factors for PEP in Multivariate Analysis

Multivariate analysis identified several independent risk factors for PEP. Pancreatic cancer was associated with a significantly lower risk (OR 0.20, 95% CI 0.07–0.57, *p* < 0.01). In contrast, pancreatography (OR 2.76, 95% CI 1.31–5.80, *p* < 0.01), use of advanced cannulation techniques (OR 2.23, 95% CI 1.18–4.20, *p* = 0.01), and metallic stent placement (OR 4.98, 95% CI 1.79–13.9, *p* < 0.01) were significantly associated with increased risk. Other variables—including age, sex, bile duct stones, endoscopic sphincterotomy, endoscopic papillary balloon dilation, peroral cholangioscopy, plastic stent placement, and pancreatic stent placement—were not significantly associated with PEP (Table 4).

**TABLE 4:** Multivariate analysis of risk factors for post‐endoscopic retrograde cholangiopancreatography (post‐ERCP) pancreatitis.

Factors	OR (95% CI)		*p*‐Value
Age (≥72 years)	0.95	(0.55–1.65)	*p* = 0.87
Male	0.77	(0.45–1.32)	*p* = 0.34
Pancreatic cancer	0.20	(0.07–0.57)	*p* < 0.01
Bile duct stone	1.48	(0.69–3.14)	*p* = 0.31
Pancreatography	2.76	(1.31–5.80)	*p* < 0.01
Advanced cannulation techniques	2.23	(1.18–4.20)	*p* = 0.01
EST	0.70	(0.38–1.29)	*p* = 0.26
EPBD	1.15	(0.31–4.23)	*p* = 0.83
POCS	2.41	(0.79–7.34)	*p* = 0.12
Biliary plastic stent	1.32	(0.65–2.67)	*p* = 0.44
Biliary metallic stent	4.98	(1.79–13.9)	*p* < 0.01
Pancreatic stent	0.39	(0.11–1.38)	*p* = 0.14

Abbreviations: CI, confidence interval; EPBD, endoscopic papillary balloon dilation; EST, endoscopic sphincterotomy; OR, odds ratio; POCS, peroral cholangioscopy.

## 4 Discussion

In this large single‐center cohort of 1,000 patients with an accessible naïve papilla undergoing ERCP for biliary diseases, we demonstrated that an overall high cannulation success rate of 97.8% can be achieved through the stepwise application of advanced cannulation techniques when initial WLC fails. While primary biliary cannulation with conventional WLC was successful in approximately 70% of cases, subsequent use of advanced methods, including DGW, precut techniques, and EUS‐RV, substantially increased the overall technical success. Regarding safety, the overall AE rate was 7.5%. However, the use of advanced cannulation techniques significantly increased the AE rate compared with WLC alone (13.3% vs. 5.2%, *p* < 0.001). Multivariate analysis further confirmed that advanced cannulation techniques were an independent risk factor for PEP, with an OR of 2.23 (95% CI, 1.18–4.20).

Previous studies have reported that biliary cannulation in patients with a naïve papilla is achieved in more than 95% of cases by expert endoscopists, whereas the rate ranges between 80% and 90% for trainees who are not yet deemed competent in this skill [10]. Consistent with these reports, our stepwise application strategy—from WLC to advanced techniques—resulted in a very high overall cannulation success rate of 97.8% in 1,000 patients with a naïve papilla. Among the advanced methods, EUS‐RV demonstrated particularly high technical success, with a success rate of 97.0% (33/34), although its use remained relatively uncommon (3%) even in the era of EUS‐BD. Other studies have reported similar findings. A retrospective study by Holt et al. [11] evaluated the frequency of EUS‐BD during ERCP and showed that biliary cannulation was successful in 99.4% (515/518) of patients with an endoscopically accessible naïve papilla, with EUS‐BD required in only 0.6% (3/524). The authors concluded that advanced ERCP techniques ensure high technical success, while the need for EUS‐BD remains rare, despite its established role in selected situations. Similarly, Nakai et al. [12] reported a cannulation success rate of 95.6% (475/497) in therapeutic ERCP for patients with a naïve papilla, with only 22 patients (4.4%) requiring rescue procedures, including EUS‐BD. Taken together with the current findings, these studies suggest that biliary cannulation success rates during ERCP for biliary diseases in patients with an accessible naïve papilla remain very high. The need for EUS‐BD as a rescue procedure is relatively uncommon, although its indication is expected to expand further as the technique continues to mature and gain wider acceptance in clinical practice.

Regarding safety, the overall AE rate was 7.5% (75/1,000), with PEP being the most common complication. A systematic review of 21 prospective studies including 16,855 patients reported an AE rate of 6.85% (95% CI, 6.46–7.24%), with PEP as the most frequent event at an incidence of 3.47% (95% CI, 3.19–3.75%) [13]. The AE rate in our study was within the range reported in previous large studies, although AE rates are influenced by various factors such as patient demographics, indications, cannulation methods, and therapeutic interventions. In our analysis, the overall AE rate was significantly higher in patients requiring advanced cannulation techniques compared with WLC alone (13.3% vs. 5.2%, *p* < 0.001). Multivariate analysis further identified the application of advanced cannulation techniques, pancreatography, and metallic stent placement as independent risk factors for PEP, which was the most common AE. These findings are consistent with previous reports. Meta‐analyses have already established precut techniques, difficult biliary cannulation, pancreatography, and self‐expandable metallic stents as significant risk factors for PEP [14, 15, 16]. Therefore, during biliary cannulation in ERCP for biliary diseases, especially when advanced cannulation techniques are required, careful attention and close monitoring are essential to minimize the risk of PEP and other procedure‐related AEs.

The timing of applying advanced cannulation techniques is also an important consideration. In the present study, both precut techniques, TPS and NKP, were applied only when WLC or DGW failed to achieve biliary cannulation after a certain period, typically around 15 min. A retrospective study by Tanikawa et al. [17], which evaluated the optimal timing for applying precut techniques during ERCP, suggested that 12.5 min was the ideal cutoff. In their comparison of cohorts undergoing precut before and after 12.5 min, the incidence of PEP was significantly lower in the optimized‐timing group than in the delayed group (5.1% vs. 13.1%, *p* < 0.05), and this result was confirmed by multivariate analysis (OR 3.13, *p* < 0.05). Furthermore, a randomized controlled trial by Maharshi et al. [18] comparing primary NKP (*n* = 151) with early NKP (*n* = 152; after two cannulation attempts) demonstrated that primary NKP significantly reduced the incidence of PEP (0.67% vs. 5.2%, *p* = 0.04) and shortened the biliary cannulation time (7.2 ± 1.7 vs. 13.8 ± 2.2 min; *p* < 0.001), although the overall success rates for biliary cannulation were similar between the groups (98.6% vs. 98.0%, *p* = 1.0). Taken together, these findings indicate that the timing and role of precutting in the algorithm for biliary cannulation warrant further investigation to establish strategies that can optimize both efficiency and safety [19].

EUS‐RV was indicated only after the failure of all advanced cannulation techniques in our biliary cannulation algorithm. However, accumulating evidence suggests that EUS‐RV may provide clinical outcomes comparable to or even better than those of precutting. A retrospective study by Dhir et al. [20], which compared EUS‐RV (*n* = 58) with precutting (*n* = 144) for difficult biliary cannulation defined as five failed attempts, demonstrated a significantly higher biliary cannulation success rate with EUS‐RV (98.3% vs. 90.3%, p < 0.03), while the AE rates did not differ significantly (3.4% vs. 6.9%, *p* = 0.27). Subsequently, Dhir et al. [21] conducted a randomized controlled trial comparing EUS‐RV (*n* = 104) with precutting (*n* = 104) in cases of difficult cannulation, defined as more than five failed attempts or a procedure time exceeding 5 min. The results showed no significant differences between the two groups in cannulation success rate (97.1% vs. 93.3%, *p* = 0.33), overall AE rate (5.8% vs. 11.5%, *p* = 0.14), or PEP incidence (1.9% vs. 8.7%, *p* = 0.06). However, the procedure time was significantly longer with EUS‐RV (47 vs. 27 min, *p* < 0.001). These findings suggest that while EUS‐RV is currently positioned as a last‐line strategy, it may warrant earlier integration into the cannulation algorithm. Further prospective studies are needed to define its optimal timing and role relative to other advanced techniques.

This study has several limitations. First, its retrospective design and single‐center setting may limit the generalizability of the findings. Second, because of its retrospective nature, decisions regarding the timing of advanced techniques were left to the discretion of the endoscopist, potentially introducing selection bias. Third, the retrospective design limited the availability of certain data, such as cannulation time, the number of cannulation attempts, and the timing of operator exchange, which may have resulted in information bias for some important variables. Finally, long‐term outcomes beyond the immediate procedural period were not assessed.

In conclusion, this study demonstrated that high biliary cannulation success rates can be achieved in patients with a naïve papilla by a structured stepwise approach that incorporates advanced techniques such as DGW, precut, or EUS‐RV. While advanced techniques were associated with a higher risk of AEs, their careful application at an appropriate stage in the algorithm can maximize both safety and efficacy. Our findings reaffirm the clinical value of a stepwise strategy and provide additional insights into the risk factors for PEP. In the future, earlier integration of advanced cannulation techniques, along with the expanding role of EUS‐BD, may further optimize outcomes and efficiency in biliary interventions.

## Author Contributions

**Takuji Iwashita** reviewed the manuscript. **Akihiko Senju** performed data collection, analysis, and drafted the manuscript. **Takuya Koizumi**, **Yosuke Ohashi**, **Shota Iwata**, **Akinori Maruta**, **Shinya Uemura**, and **Masahito Shimizu** were involved in patient care.

## Conflicts of Interest

Takuji Iwashita is a Deputy Editor‐in‐Chief of DEN OPEN. The other authors disclosed no conflicts of interest.

## Funding

No funding was provided for this study.

## Ethics Statement

The study protocol was approved by the Institutional Review Board of Gifu University Hospital (2025‐179)

## Consent

N/A

## Clinical Trial Registration

N/A
